# Supplementary figures and images for: Chromosome engineering points to the cis‐acting mechanism of chromosome arm‐specific telomere length setting and robustness of plant phenotype, chromatin structure and gene expression
Source: Plant J. 2025 Feb 17;121(4):e70024. doi: 10.1111/tpj.70024 (PMC11832813; doi:10.1111/tpj.70024)

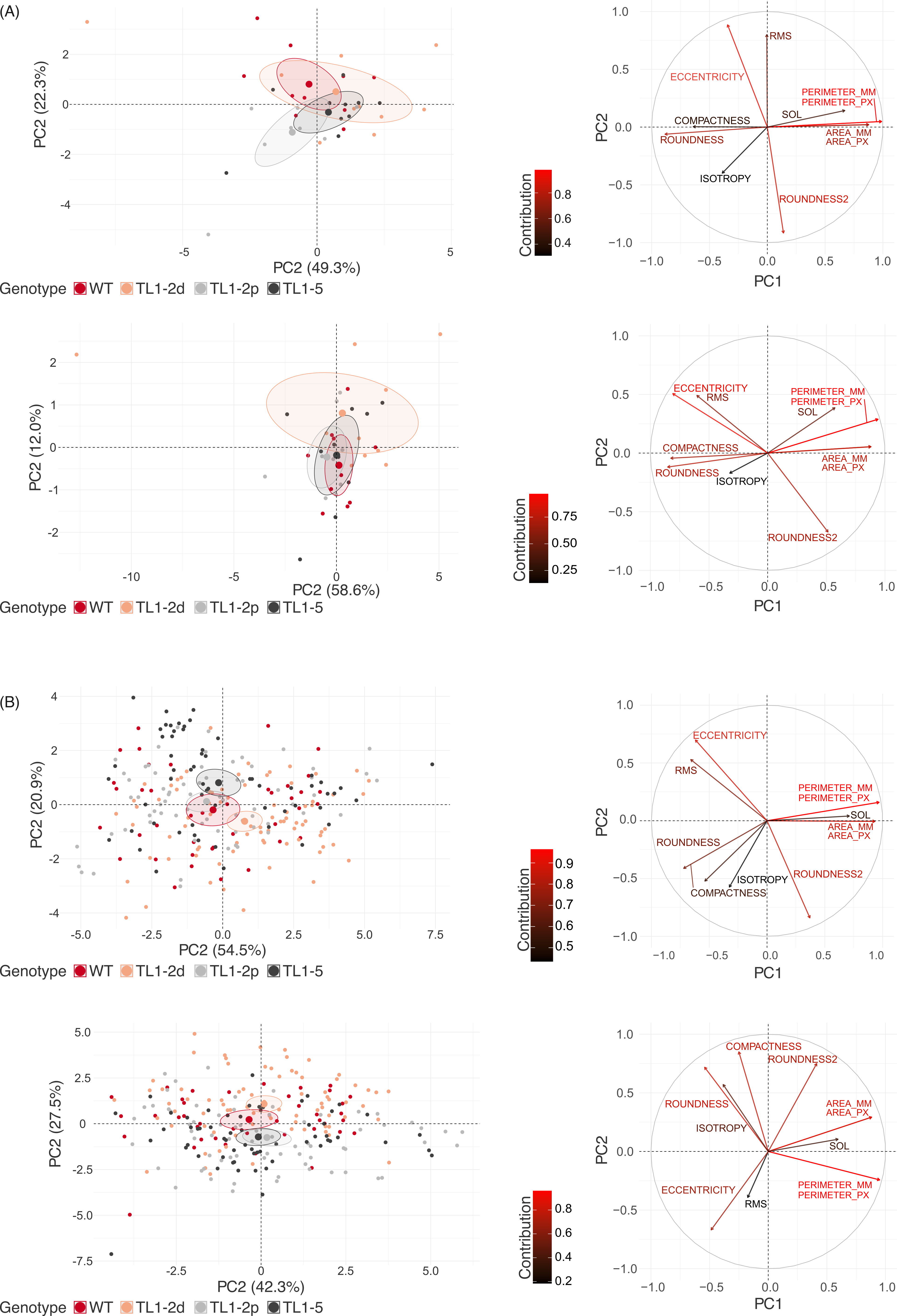

Supplement: Supplementary file 1 — Figure S1. Visualization of RGB‐imaging‐based parameters as PCA plots (left) and bi‐plots (right) of plants from T4 (a) and T6 (b) generations at 4 WAS (upper panels) and 5 WAS (lower panels). The length of the arrow (the longer the arrow, the higher the contribution) explains the contribution of tested variables. The differences in plant genotypes are indicated in PCA plots as centroids with ellipses representing their 95% confidence interval. [file TPJ-121-0-s004.tif]

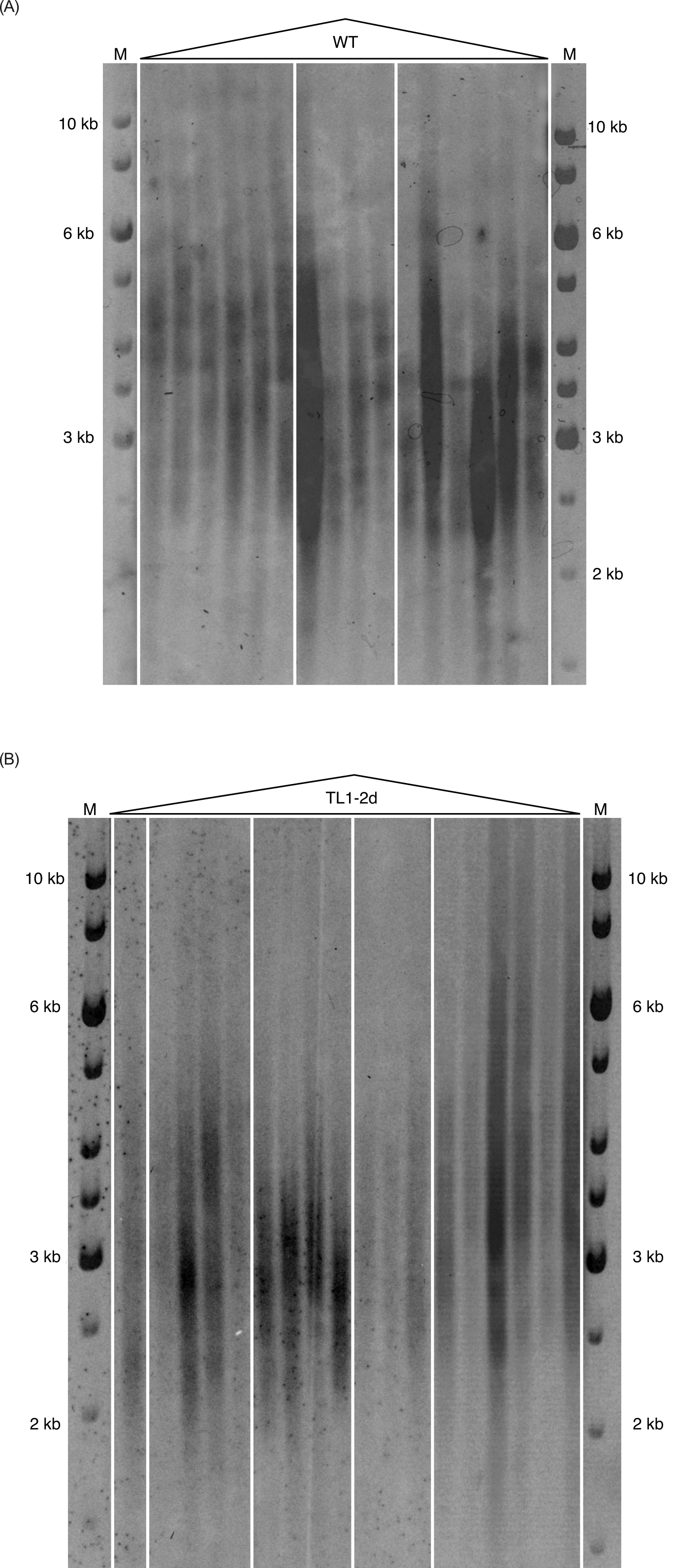

Supplement: Supplementary file 2 — Figure S2. Telomere lengths analyzed in leaves of T5 plants by the terminal restriction fragments (TRF) method. (a) WT plants. (b) Plants with translocated arms of chromosomes 1 and 2 with deletion at the junction site, TL1‐2d. (c) Plants with translocated arms of chromosomes 1 and 2 with perfect ligations at both junction sites, TL1‐2p. (d) Plants with translocated arms of chromosomes 1 and 5, TL1‐5. Each lane represents an individual plant (i.e., biological replicate). M, DNA size marker, 1‐kb DNA Gene Ruler Ladder, Thermo Fisher Scientific. Telomere‐specific hybridization signals were evaluated by the WALTER toolset (Lycka et al., 2021) (Figure S3a). [file TPJ-121-0-s003.zip › Figure S2_A+B.tif]

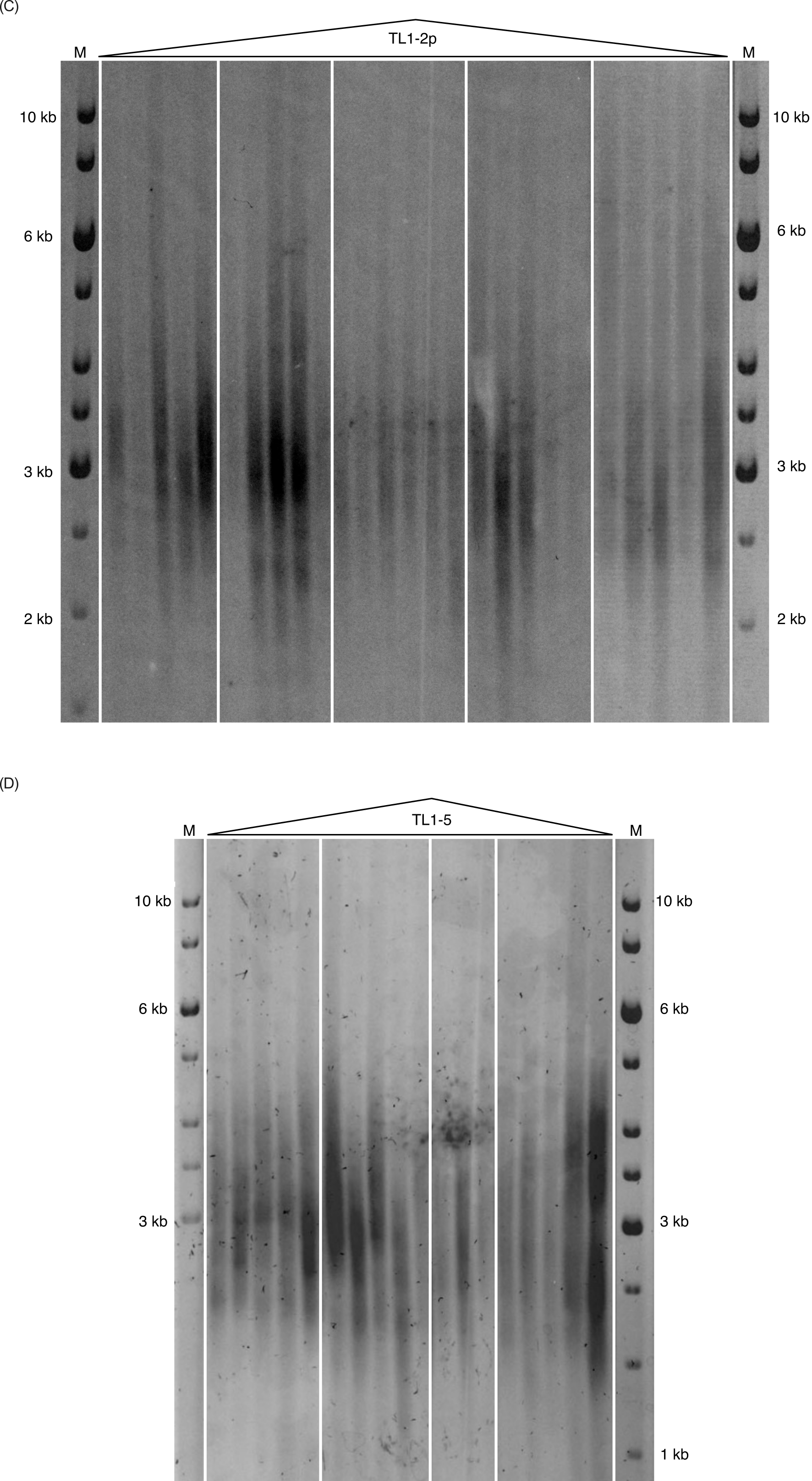

Supplement: Supplementary file 2 — Figure S2. Telomere lengths analyzed in leaves of T5 plants by the terminal restriction fragments (TRF) method. (a) WT plants. (b) Plants with translocated arms of chromosomes 1 and 2 with deletion at the junction site, TL1‐2d. (c) Plants with translocated arms of chromosomes 1 and 2 with perfect ligations at both junction sites, TL1‐2p. (d) Plants with translocated arms of chromosomes 1 and 5, TL1‐5. Each lane represents an individual plant (i.e., biological replicate). M, DNA size marker, 1‐kb DNA Gene Ruler Ladder, Thermo Fisher Scientific. Telomere‐specific hybridization signals were evaluated by the WALTER toolset (Lycka et al., 2021) (Figure S3a). [file TPJ-121-0-s003.zip › Figure S2_C+D.tif]

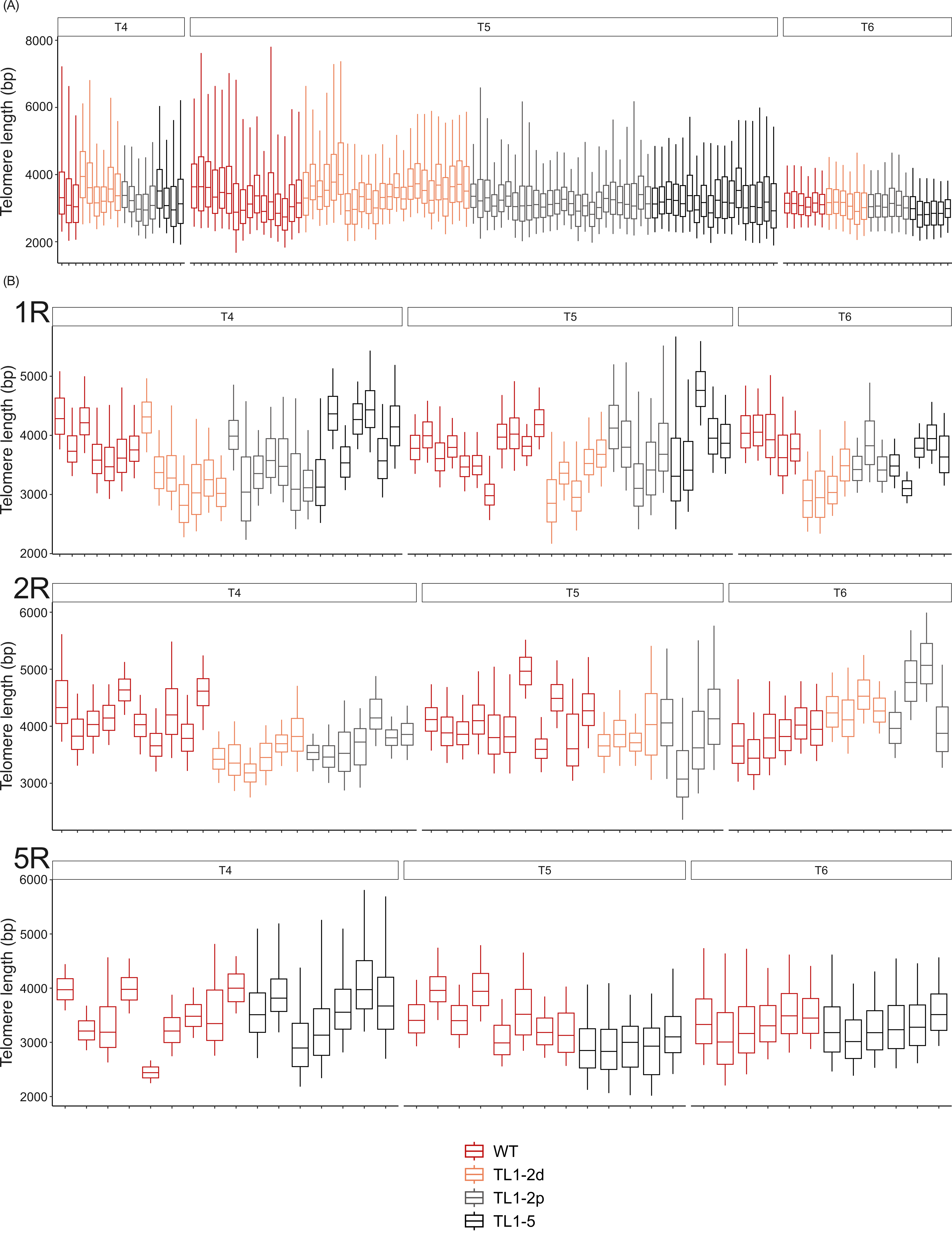

Supplement: Supplementary file 3 — Figure S3. Distribution of telomere lengths in WT plants and plants with translocated chromosome arms. Telomere‐specific hybridization signals obtained in analyses of telomere lengths by TRF (a) and PETRA (b) protocols were evaluated using the WALTER toolset (Lycka et al., 2021) and presented as boxplots. The central line indicates the weighted median, box limits represent the first and third quartiles and whiskers indicate the minimum and maximum of the selected area of telomeres. (a) Evaluation of hybridization signals of bulk telomeres obtained in analyses of T4, T5 and T6 generations of plants with translocated chromosome arms by the TRF protocol. For raw data of plants of T5 generation, see Figure S2. (b) Evaluation of hybridization signals of individual chromosome arms (1R, 2R, 5R) obtained in analyses of T4, T5 and T6 generations of plants with translocated chromosome arms by the PETRA protocol. For raw data of plants of T5 generation, see Figure S4. [file TPJ-121-0-s007.tif]

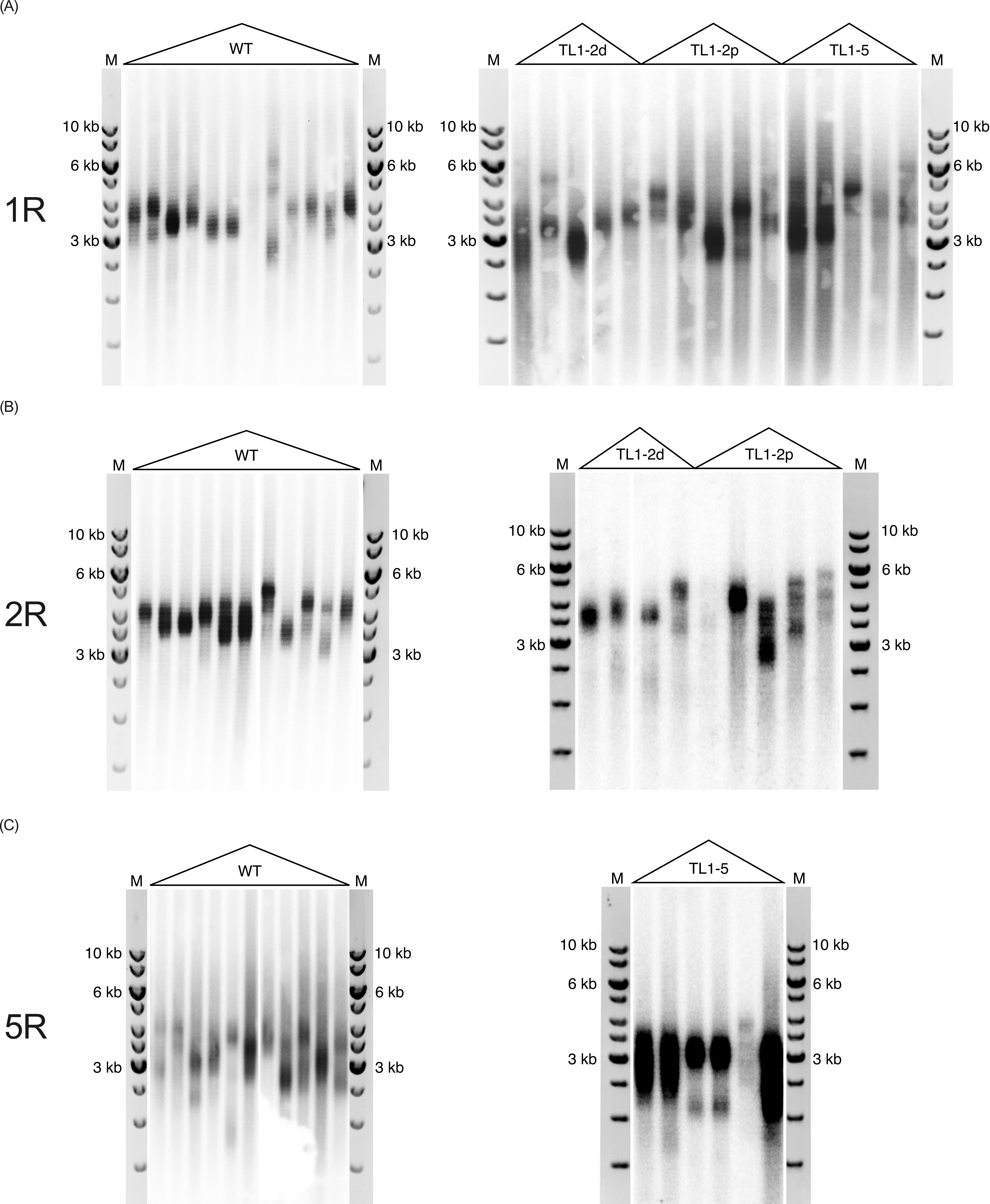

Supplement: Supplementary file 4 — Figure S4. Telomere lengths at individual chromosome arms analyzed in leaves of T5 plants by the primer extension telomere repeat amplification (PETRA) method. (a) Telomeres at the 1R chromosome arms were analyzed in WT plants, plants with translocated arms of chromosomes 1 and 2 with deletion at the junction site (TL1‐2d), plants with translocated arms of chromosomes 1 and 2 with perfect ligations at both junction sites (TL1‐2p), and plants with translocated arms of chromosomes 1 and 5 (TL1‐5). (b) Telomeres at 2R chromosome arms were analyzed in WT, TL1‐2d and TL1‐2p plants. (c) Telomeres at 5R chromosome arms were analyzed in WT and TL1‐5 plants. Each lane represents an individual plant (i.e., biological replicate). M, DNA size marker, 1‐kb DNA Gene Ruler Ladder, Thermo Fisher Scientific. Telomere‐specific hybridization signals were evaluated by the WALTER toolset (Lycka et al., 2021) (Figure S3b). [file TPJ-121-0-s006.tif]
